# Silver nanoparticle functionalized by glutamine and conjugated with thiosemicarbazide induces apoptosis in colon cancer cell line

**DOI:** 10.1038/s41598-024-54344-x

**Published:** 2024-02-15

**Authors:** Hadi Taati, Helia Sangani, Arash Davoudi, Samira Safabakhsh Kouchesfahani, Mohammad Hedayati, Sana Tarashandeh Hemmati, Taraneh Ghasemipour, Shahrzad Aghajani, Mahan Farah Andooz, Maryam Amanollahi, Fakhrieh Kalavari, Ali Salehzadeh

**Affiliations:** 1 Department of Biology, Rasht Branch, Islamic Azad University, Rasht, Iran; 2 Department of Biology, Tonekabon Branch, Islamic Azad University, Tonekabon, Iran; 3 Guilan University of Medical Sciences, Rasht, Iran; 4 Department of Biology, Tehran Medical Sciences, Islamic Azad University, Tehran, Iran; 5 Department of Pathology, Guilan University of Medical Sciences, Rasht, Iran

**Keywords:** Apoptosis, Colon cancer, Flow cytometry, Silver nanoparticles, Thiosemicarbazide, Cancer, Cell biology, Molecular medicine, Nanoscience and technology

## Abstract

The high mortality rate of colon cancer indicates the insufficient efficacy of current chemotherapy. Thus, the discussion on engineered metal nanoparticles in the treatment of the disease has been considered. In this study, silver nanoparticles were functionalized with glutamine and conjugated with thiosemiccarbazide. Then, anticancer mechanism of Ag@Gln-TSC NPs in a colon cancer cell line (SW480) was investigated. Characterizing Ag@Gln-TSC NPs by FT-IR, XRD, EDS-mapping, DLS, zeta potential, and SEM and TEM microscopy revealed that the Ag@Gln-TSC NPs were correctly synthesized, the particles were spherical, with surface charge of − 27.3 mV, high thermal stability and low agglomeration level. Using MTT assay we found that Ag@Gln-TSC NPs were significantly more toxic for colon cancer cells than normal fibroblast cells with IC_50_ of 88 and 186 µg/mL, respectively. Flow cytometry analysis showed that treating colon cancer cells with Ag@Gln-TSC NPs leads to a considerable increase in the frequency of apoptotic cells (85.9% of the cells) and increased cell cycle arrest at the S phase. Also, several apoptotic features, including hyperactivity of caspase-3 (5.15 folds), increased expression of *CASP8* gene (3.8 folds), and apoptotic nuclear alterations were noticed in the nanoparticle treated cells. Furthermore, treating colon cancer cells with Ag@Gln-TSC NPs caused significant down-regulation of the HULC Lnc-RNA and PPFIA4 oncogene by 0.3 and 0.6 folds, respectively. Overall, this work showed that Ag@Gln-TSC NPs can effectively inhibit colon cancer cells through the activation of apoptotic pathways, a feature that can be considered more in studies in the field of colon cancer treatment.

## Introduction

Colon cancer is known as the fifth most common cancer in the world. It is estimated that about 0.6 million patients die every year due to this disease, which is the fifth cause of death from cancer^1^. In the early stages of the disease, surgical resection is considered as the first treatment option. In these cases, chemotherapy is considered a post-surgery adjuvant treatment in order to reduce the risk of relapse. The main treatment of this disease in its advanced stages (III and IV) is based on the use of anti-cancer drug regimens, which aim to control disease development and destroy metastatic masses. The high death rate of colon cancer is mostly related to the involvement of lymph nodes and the occurrence of metastatic disease^2^. This shows the insufficient efficiency of the current chemotherapy drugs in the treatment of this disease in its advanced phases.

The extensive growth of nanotechnology toward the development of nanomedicine agents has created significant hopes in the field of cancer diagnosis and treatment. Due to their small size and large surface area, nanoparticles have specific features that make them promising tools in the field of cancer diagnosis and treatment^3^. Metallic nanoparticles possess unique properties which makes them attractive candidates for various applications especially in field of experimental medicine and drug delivery^4^. Currently, a large number of metal nanoparticles are being studied to be used in the treatment of different types of cancer. However, lack of specificity, insufficient efficacy, and systemic toxicity are the most important obstacles to using these compounds in clinical trials^3^. In order to overcome these limitations, surface modification of nanoparticles has been introduced as a promising solution, so many researchers focus on the design of multifunctional nanomedicines with the aim of improving biocompatibility and anticancer efficacy, and providing targeted drug delivery systems^5^.

Silver nanoparticles (Ag NPs) have been largely investigated in biology and medicine. AgNPs play an efficient role against a variety of cancer both in vitro and in vivo, including cervical cancer, breast cancer, lung cancer, hepatocellular carcinoma, nasopharyngeal carcinoma, hepatocellular carcinoma, glioblastoma, colorectal adenocarcinoma, and prostate carcinoma^6^. The AgNPs can play an important role in engineering with anticancer drugs with maximum therapeutic effect^7^. Some possible mechanisms involving the anticancer effects of AgNPs have been proposed. AgNPs can cause apoptosis or necrosis by destroying the ultrastructure of cancer cells, inducing ROS production and DNA damage, disruption mitochondrial respiratory chain and ATP synthesis, inactivating enzymes, as well as regulating signaling pathways^8–10^. Compared with other metal nanoparticles, Ag NPs have better biocompatibility with mammalian cells, which makes them promising candidates to be used in cancer diagnosis and chemotherapy^11^.

Thiosemicarbazones and their metal complexes present a wide range of applications that stretch from their use in analytical chemistry, through pharmacology to nuclear medicine. The presence of amide, imine and thione groups makes them potential polydentate ligands and it is not surprising that numerous thiosemicarbazone complexes have been prepared and characterized. In addition, in the last few years there has been a growing attention towards thiosemicarbazones related to their range of biological properties, specifically as antifungal, antiviral, antibacterial and anticancer agents^12^. Thiosemicarbazones have been gaining considerable attention for its cytotoxic effects on various cancer cell lines. It has been reported that thiosemicarbazones could drive cell apoptosis through the inhibition of DNA replication as well as the generation of oxidative stress. Additionally, it has been found that thiosemicarbazone derivatives could interrupt the Iron acquisition mechanism which in turn disrupts normal metabolic processes of the cell^13,14^. These findings suggest that Thiosemicarbazones may hold promise as a novel anticancer agent.

Recent studies have shown that functionalization of metal nanoparticles with biocompatible molecules, such as glutamine, could improve the biocompatibility of the nanoparticles and also facilitate their conjugation with different therapeutic compounds^15,16^. Considering the anticancer properties of silver nanoparticles and thiosemicarbazide, the current work investigates the anticancer effect of silver nanoparticles functionalized by Glutamine and conjugated with thiosemicarbazide (Ag@Gln-TSC NPs) on a colon cancer cell line and evaluates the expression of the *CASPS8, HULC,* and *PPFIA4* genes in nanoparticle treated cells.

## Materials and methods

### Synthesis of nanoparticles

To synthesize Ag NPs, a 150 mL of 5 mM solution of AgNO_3_ was prepared and pH was adjusted to 11.0 using 10% NaOH solution. The mixture was heated in an oil bath at 80 °C for 2 h. The precipitate was collected by centrifugation, washed with distilled water and ethanol, and finally, dried at 70 °C for 8 h. For functionalization with glutamine, at first, a 150 mL suspension containing 300 mg of Ag NPs and 152 mg of glutamine was prepared; the pH was adjusted at 11.0 using 10% NaOH solution and heated at 80 °C for 2 h. The resulting Ag@Gln NPs were harvested, rinsed with distilled water and 96% ethanol, and dried at 70 °C. To synthesize Ag@Gln-TSC NPs, 500 mg of Ag@Gln and 200 mg of thiosemicarbazide were suspended in 150 mL of 96% ethanol and maintained in a 40 °C water bath for 24 h. Next, the nanoparticles were collected by centrifugation, rinsed, and dried at 70 °C for 8 h^17^.

### Physicochemical characterization of nanoparticles

The functional groups of Ag@Gln, and Ag@Gln-TSC, in a range of 500–4000 cm^−1^ were investigated by an FT-IR assay using a Spectrum Two, Perkin Elmer FT-IR device. XRD analysis was used to characterize the crystal structure of Ag@Gln-TSC NPs using Co-Ka X-radiation at k = 1.79 Å. Morphological characteristics and elemental composition of the Ag@Gln-TSC nanoparticles were evaluated by Field Emission Scanning Electron Microscopy (FE-SEM) (Zeiss- Sigma VP model) and Transmission Electron Microscopy (TEM) (Zeiss EM-10C-100 kV). Also, zeta potential and DLS analyses were conducted to characterize the surface charge and hydrodynamic size of the Ag@Gln-TSC NPs by Malvern Zeta Sizer instruments (Malvern Ltd, 6.32). Furthermore, the thermal stability of the particles was characterized by thermogravimetric analysis (TGA) (Rhemometric Scientific, STA 1500, USA).

### Cell lines

To characterize the anticancer effect of Ag@Gln-TSC NPs, the SW480 colon cancer and HEK293 cell lines were purchased from the Cell Bank of Pasteur Institute of Iran. Cell culture was performed in 25 cm^3^ cell culture flasks in RPMI 1640 medium enriched with 10% fetal bovine serum and penicillin–streptomycin.

### Cell viability assay

The MTT assay was used to characterize cytotoxicity effect of Ag@Gln-TSC NPs on colon cancer and normal cell lines. In brief, about 10,000 cells were propagated in a 96-well culture plate to reach the 50% confluency and treated with different quantities of Ag@Gln-TSC NPs in a range of 15.625–500 µg/mL. A few wells that did not contain nanoparticles were considered as controls. Following incubation at 37 °C for 24 h, 0.2 mL of MTT (2-(4,5-dimethythiazol-2-yl)-2,5-diphenyltetrazolium bromide) solution (Sigma-Aldrich, USA) was added to the wells and incubated for further 4 h. Then, the wells were emptied and 0.2 mL of DMSO (Sigma-Aldrich, USA) was added and after 30 min the OD_570_ was measured (Bio-Rad, Hercules microplate reader,). The inhibition percentage due to exposure to nanoparticles was determined based on the following formula^18,19^:

$$Inhibition \left( {\text{\% }} \right) = \frac{Abs \;of\; control - Abs \;of\; Test}{{Abs \;of\; control}} \times 100$$

### ROS level

To investigate the effect of treatment with Ag@Gln-TSC NPs on ROS generation, colon cancer cells (5 × 10^5^) were treated with IC_50_ concentration of NP for 24 h. The content of ROS in cells was determined utilizing a ROS Assay kit (ab186027, abcam, USA) and the fluorescent probe 2,7-dichlorodihydrofluorescein diacetate (DCFH-DA) according to the instructions provided by the manufacturer. After incubation in the dark at room temperature for 60 min, the cells were washed and their fluorescence intensity was compared with control cells^20^.

### Investigating the apoptotic effect of Ag@Gln-TSC NPs

To evaluate the mechanism of cell toxicity of Ag@Gln-TSC NPs, a fluorescein iso-thiocyanate (FITC)-annexin V and propidium iodide (PI) apoptosis detection kit (Sigma-Aldrich, USA) was used. Colon cancer cells were propagated and then, 5 × 10^5^ of cells treated with the nanoparticles at their 50% inhibitory concentration (IC_50_) for 24 h, while control cells were treated with PBS. After incubation, the cells were stained with propidium iodide and Annexin V, and then, cell apoptosis/necrosis frequency was quantified by a flow cytometry device (ZE5 cell Analyzer, Bio-Rad, USA).

### Cell cycle analysis

To characterize the effect of Ag@Gln-TSC on cell cycle phases, 5 × 10^5^ of colon cancer cells was grown in 6-well plates, and treated with the nanoparticles (at their IC_50_). After incubation for 24 h, the cells were stained propidium iodide (ab287852 kit, abcam, USA) and then, treated with RNase A (100 µg/mL). Finally, cell cycle phases were determined based on the cell DNA content and compared with control cells.

### Caspase-3 activity

The activity of caspase-3 in Ag@Gln-TSC treated and control cells were quantified by the method described by Salehzadeh et al.^21^. At first, 5 × 10^5^ of SW480 cells were treated with IC_50_ concentration of NP for 24 h while control cells were treated with PBS. Next, the cells were harvested, lysed and their supernatant was treated with DEVD-pNA (CASP3 assay kit, Sigma-Aldrich, USA). Finally, the optical density of the supernatant samples was recorded at 405 nm.

### Hoechst staining

To accomplish the Hoechst nuclear staining, colon cancer cells were treated with Ag@Gln-TSC NPs for 24 h (at IC_50_ concentration) and then, stained with the Hoechst 33,258 solution (Sigma-Aldrich, USA). Subsequent, the cells were washed with PBS, examined under a fluorescent microscope, and compared with control cells (Incell Analyser 2000, USA).

### Gene expression

The expression level of the *CASP8*, *HULC*, and *PPIA4* in Ag@Gln-TSC treated cells was quantified relative to control cells. About 5 × 10^5^ of colon cancer cells were grown and treated with IC_50_ concentration of NP for 24 h. Then, the cells were collected, washed, and their total RNA content was extracted using the Thermo reagent (Thermo Fisher Scientific, USA), according to the instruction. Next, cDNA molecules were synthesized using the extracted RNA templates by Yekta Tajhiz’s (Iran) cDNA synthesis kit, according to the manufacturer’s instruction.

Finally, the relative expression of the *CASP8*, *HULC*, and *PPIA4* genes in nanoparticle-treated cells was quantified by Real-Time PCR using gene-specific primers (Table 1). Gene expression changes were calculated using the 2^−ΔΔct^ method and the *GAPDH* gene was used as the internal control gene^22^.

**Table 1:** Sequence of the primers used in this work.

Gene	Forward primer (5′–3′)	Reverse primer (5′–3′)	References
*GAPDH*	CCCACTCCTCCACCTTTGAC	CATACCAGGAAATGAGCTTGACAA	^23^
*CASP8*	GACTGGATTTGCTGATTACCTACCTAA	CCTCAATTCTGATCTGCTCACTTCT	^23^
*HULC*	ACAGACCAAAGCATCAAGCA	TTTGCCACAGGTTGAACACTT	^24^
*PPIA4*	AGAGAATTGCAGCCCTCACC	CCAGCTCCTGGTTCTTCTCC	This study

### Statistical analysis

All experiments were performed in three replicates and their significant differences were assessed using the one-way Analysis of Variance. The *p* values of less than 0.05 were considered statistically significant.

## Results

### Characteristics of the nanoparticles

According to the FT-IR analysis, the Ag-Gln NPs spectrum shows a peak at 535 cm^−1^ that seems to be related to silver atoms and also a peak at 710 cm^−1^ that is related to the C–H bond. Furthermore, the peaks at 883 and 1067 cm^−1^ are associated with the C=O and C–O bonds. In addition, the C–C, C=N, N–H, and O–H bonds resulted in some peaks at 1378, 1453, 1555, and 3634 cm^−1^, respectively.

**Figure 1:**
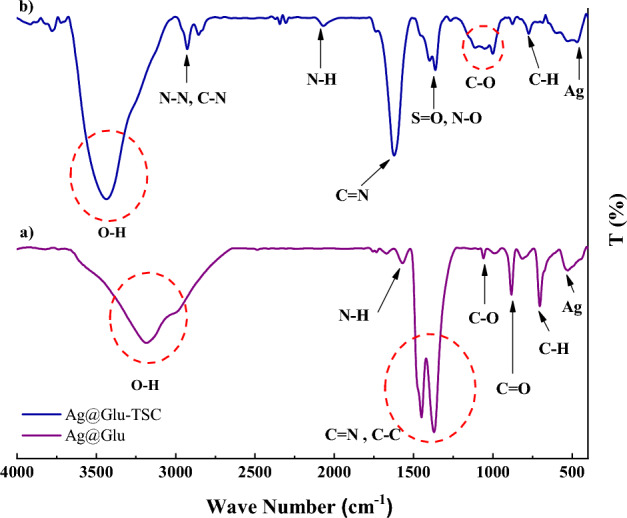
FT-IR analysis of Ag@Gln-TSC and Ag@Gln-TSC indicates correct synthesis and conjugation of the particles.

Studying the FT-IR spectrum of Ag@Gln-TSC NPs, a peak at 469.28 cm^−1^ is related to silver atom, and peaks at 773.9, 1000.3, and 1362.3 cm^−1^ are associated with C–H, C–O, S=O, and N–O bonds. In addition, the peaks at 1622.24, 2067.3, 2855.08, and 2629.41 cm^−1^ are related to the C=N, N=H, C–N, and N–N bond, respectively. The peaks that are observed in the range of 3000–4000 cm^−1^ are associated with the O–H bond (Fig. 1).

**Figure 2:**
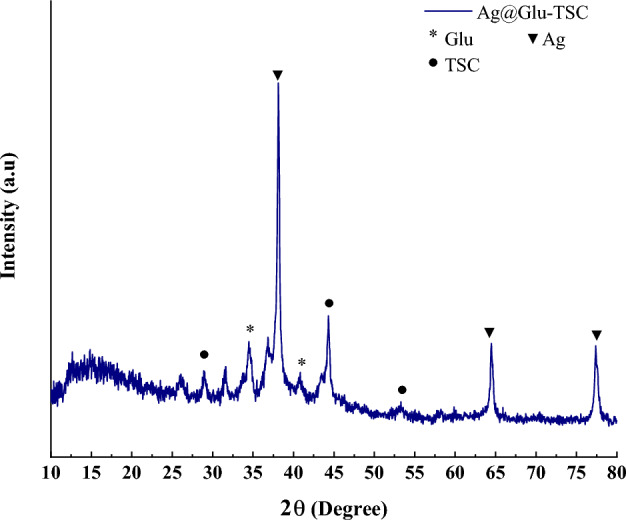
XRD analysis of Ag@Gln-TSC NPs.

According to the XRD spectrum of Ag@Gln-TSC NPs, the peaks at 2*θ* of 38.1°, 64.44°, and 77.33° are related to silver nanoparticles that comply with the JCPDS card number 0719-078-01^25^. Also, the peaks at 2*θ* of 34.44, and 40.84 are associated with glutamine molecules^26^. Furthermore, considering the amorphous nature of TSC, three relatively broad peaks are observed at 2*θ* of 28.93°, 44.29°, and 53.26° that indicate the presence of TSC in nanoparticle structure^27^. The XRD pattern of Ag@Gln-TSC NPs was displayed in Fig. 2.

**Figure 3:**
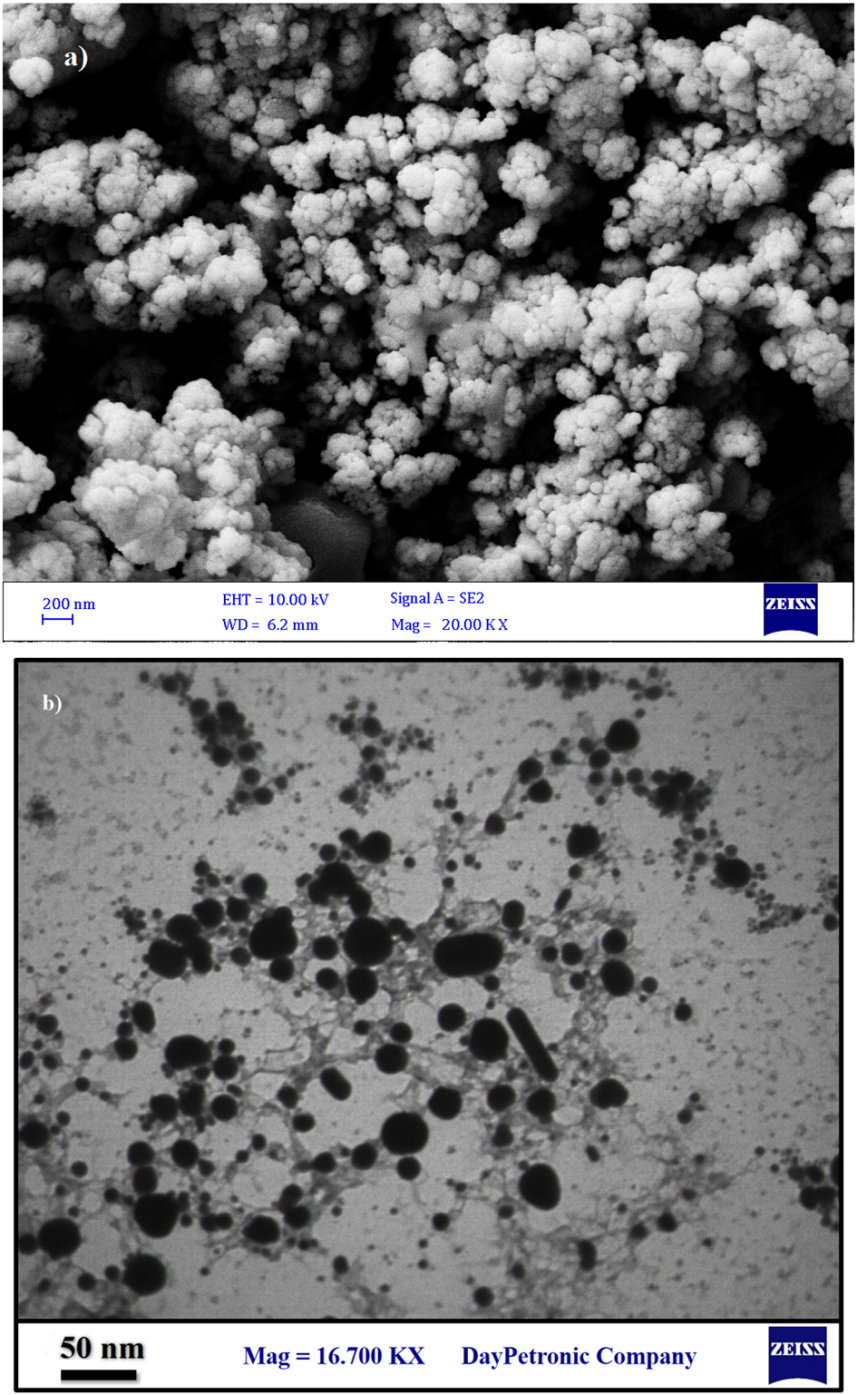
SEM (**a**) and TEM (**b**) images of Ag@Gln-TSC NPs. The nanoparticles are spherical and synthesized in a nano-scale size range.

**Figure 4:**
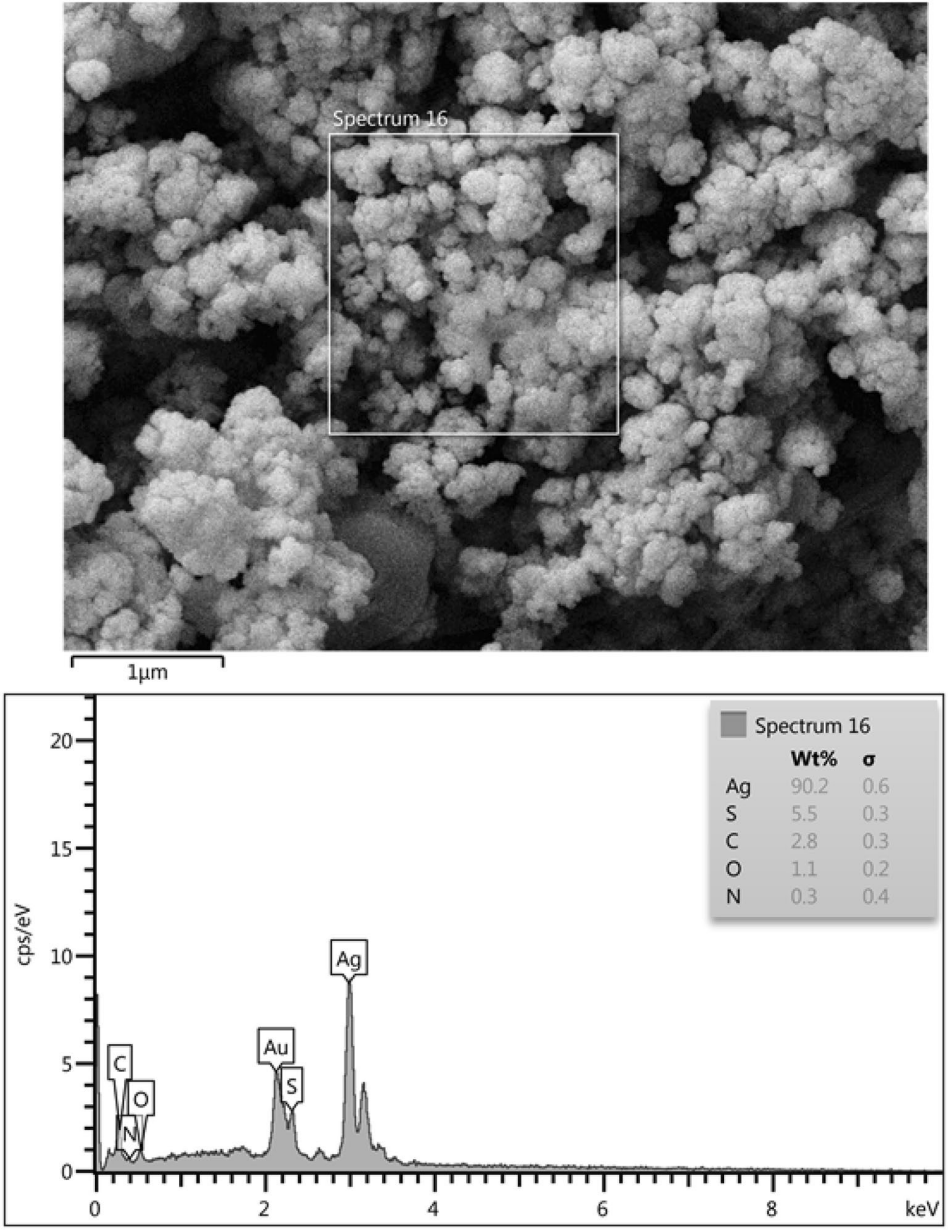
EDS-mapping analysis of Ag@Gln-TSC NPs presents the elemental composition of the particles. The particles were composed of Ag, S, C, O, and N atoms.

According to the SEM and TEM microscopy, Ag@Gln-TSC NPs were almost spherical and were synthesized in a nanoscale (Fig. 3). EDS-mapping analysis revealed that the constituent elements of the nanoparticle structure include Ag, S, C, O, and N atoms confirming the purity of the nanoparticles (Fig. 4).

**Figure 5:**
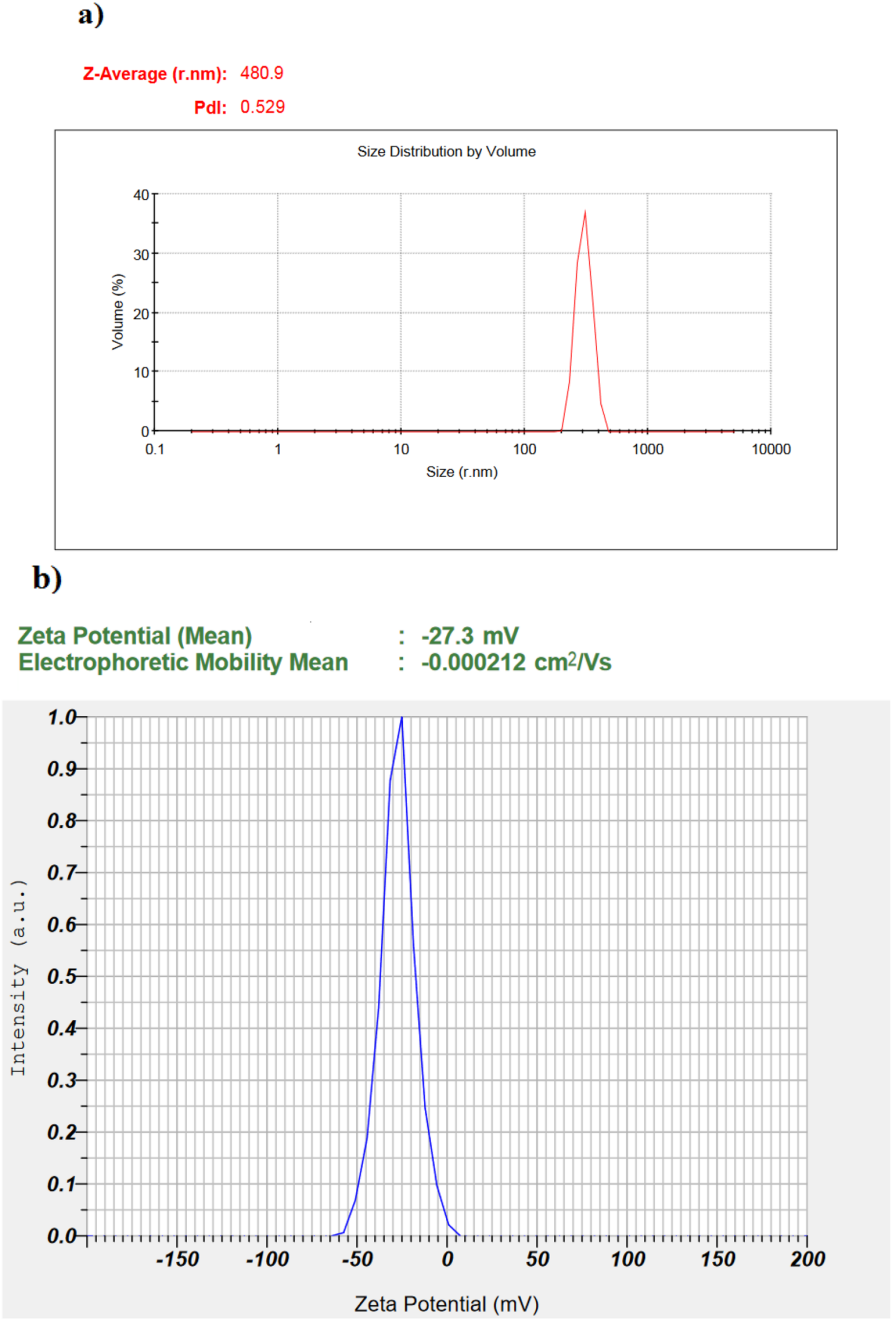
(**a**) DLS analysis and (**b**) Zeta potential of Ag@Gln-TSC NPs.

**Figure 6:**
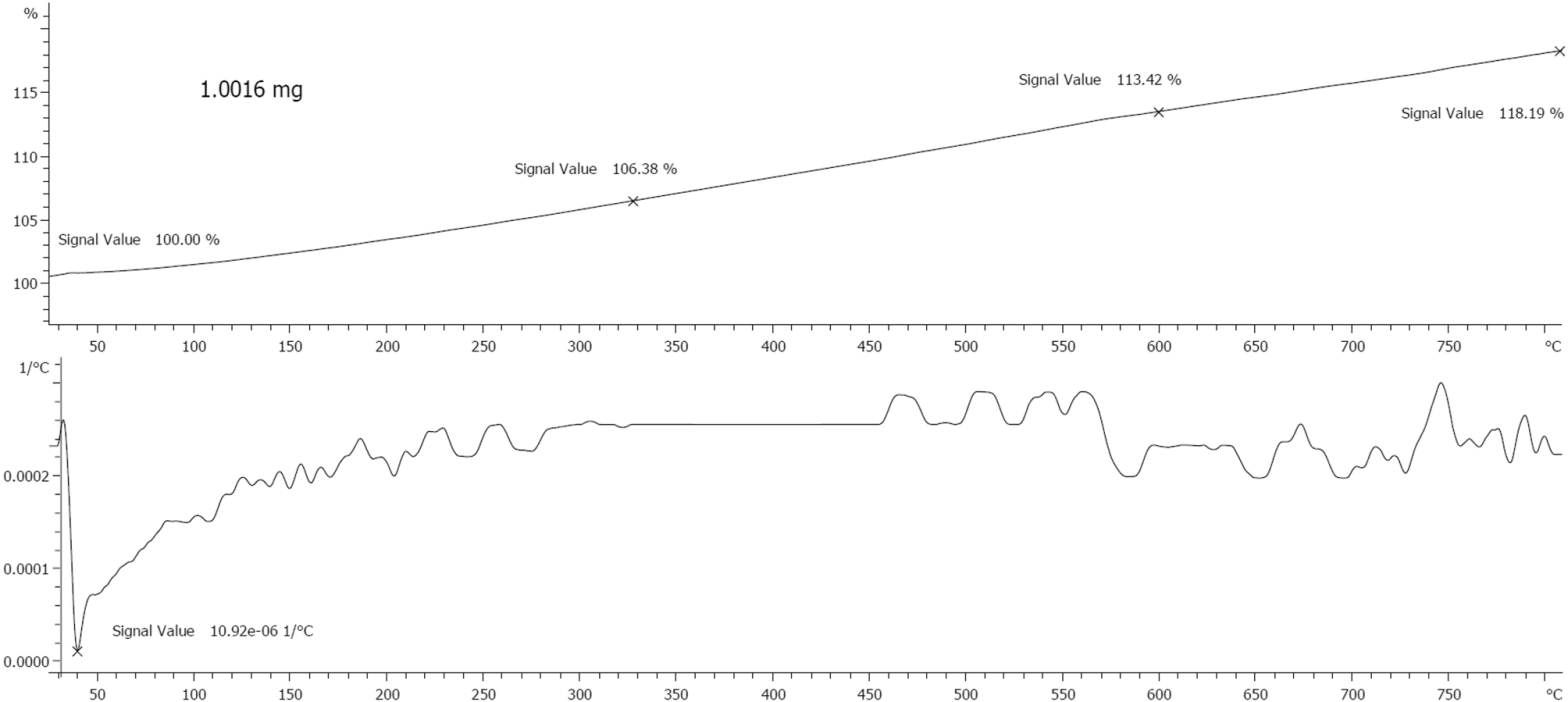
Thermal stability of Ag@Gln-TSC NPs.

According to the DLS analysis, the size of nanoparticles in an aqueous environment was in a size range of 200–500 nm and the average particle size was 480.9 nm. Also, the surface charge of the particles was − 27.3 mV, which can provide sufficient repulsive forces between particles which reduce particle agglomeration. Figure 5 displays DLS and zeta potential analysis of Ag@Gln-TSC NPs. According to the TGA analysis, Ag@Gln-TSC NPs showed considerable thermal stability so they did not show significant weight loss at temperatures up to 800 °C (Fig. 6).

### Cell viability assay

**Figure 7:**
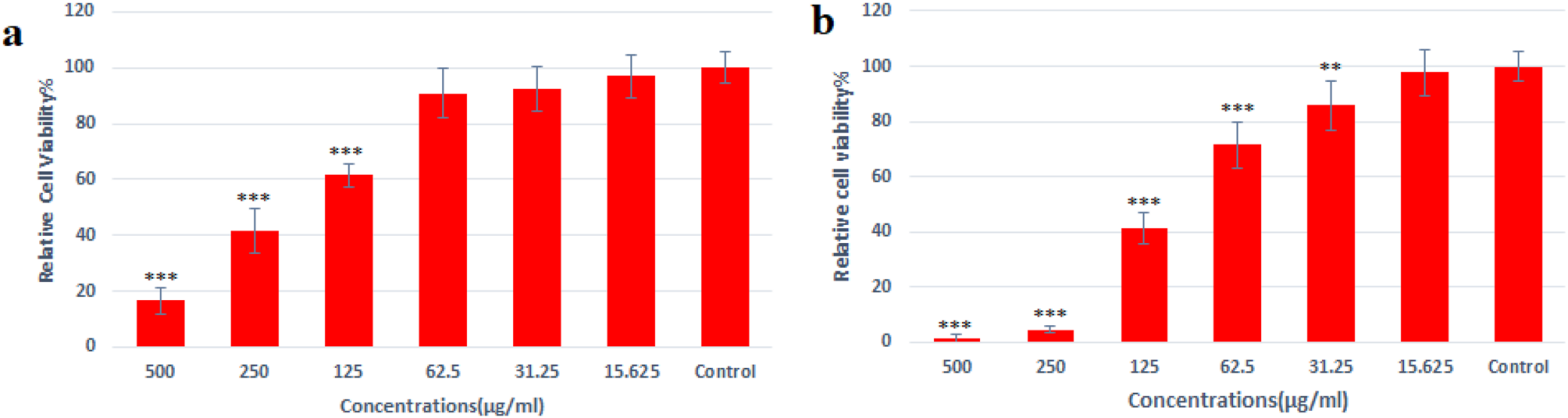
Cell viability assay. (**a**) Normal cell line and (**b**) colon cancer cell line. The results showed that Ag@Gln-TSC was more toxic for colon cancer cells than normal cells. The IC_50_ for SW480 and HEK293 cell lines were 88 and 186 µg/mL, respectively. Asterisks (*) indicate a significant difference with the control group (*P < 0.05, **P < 0.01, ***P < 0.001).

According to the MTT assay, Ag@Gln-TSC NPs have dose-dependent toxicity for colon cancer and normal cells. Treating colon cancer cells with Ag@Gln-TSC NPs at 31.25 µg/mL significantly reduced cell viability by reduced viability of SW480 and HEK293 cells by 14.2%, while at concentrations up to 62.5 µg/mL, the nanoparticles were not considerably toxic for normal cells as their viability only reduced by 9.2%. The highest inhibitory potential of Ag@Gln-TSC NPs was observed at 500 µg/mL, at which they reduced the viability of colon cancer and normal cells by 98.7 and 83.5%, respectively. The IC_50_ of the nanoparticles for colon cancer and normal cells were 88 and 186 µg/mL, respectively. Figure 7 displays the effect of Ag@Gln-TSC NPs on the viability of studied cell lines.

### ROS level

**Figure 8:**
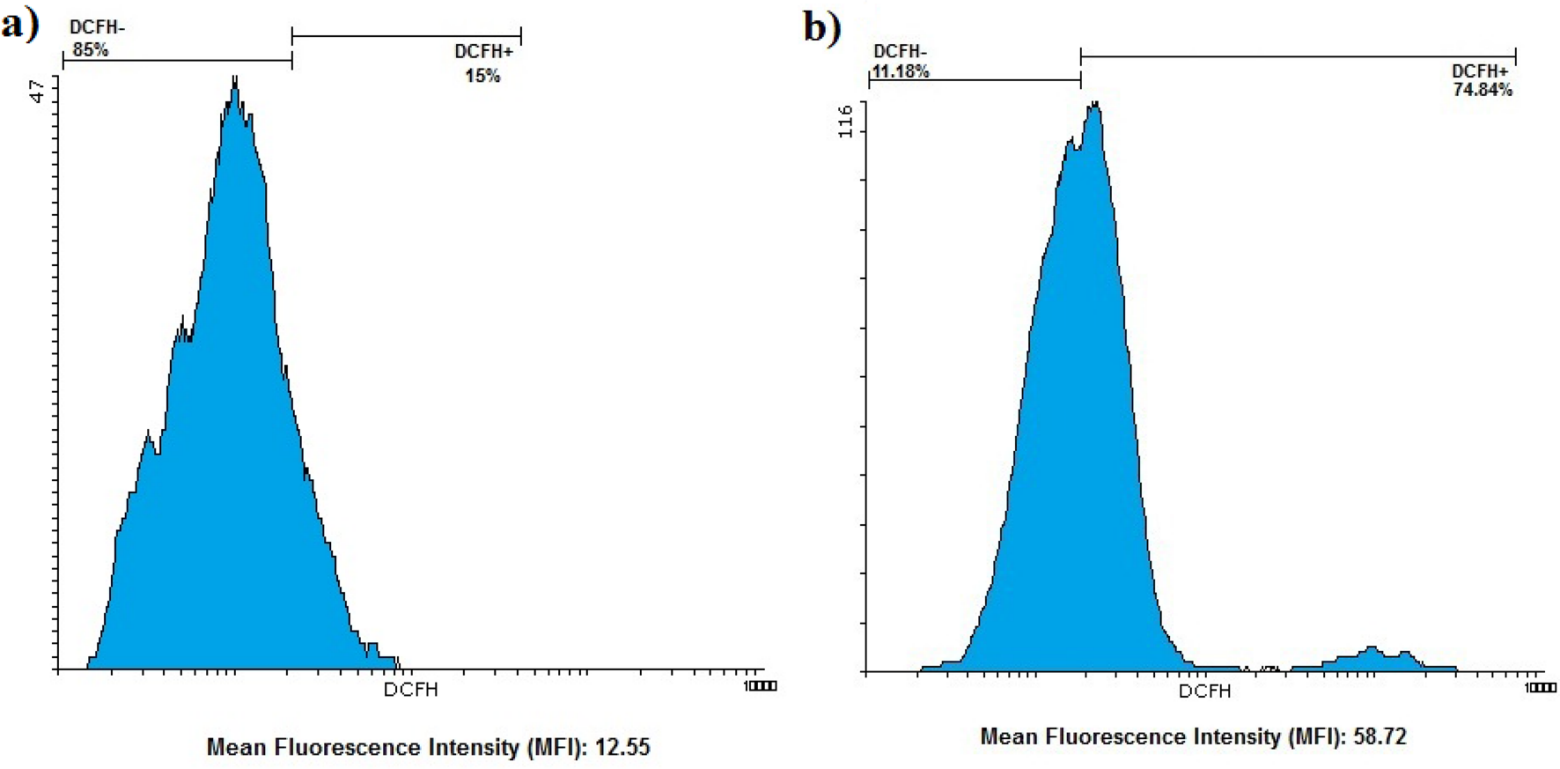
ROS level in the control (**a**) and nanoparticle-treated (**b**) groups. ROS level assay revealed that the generation of ROS molecules in Ag@Gln-TSC treated cells was considerably higher than control cells.

After treating colon cancer cells with Ag@Gln-TSC NPs, the ROS level was measured and compared with the control group. The results showed that the mean fluorescence intensity (MFI), which reflects ROS level, was considerably higher in nanoparticle-treated cells than in control cells. The MFI in nanoparticle-treated and control cells were 58.72 and 12.55, respectively. Figure 8 shows the ROS levels in nanoparticle-treated and control cells.

### Cell apoptosis and cell cycle phases

**Figure 9:**
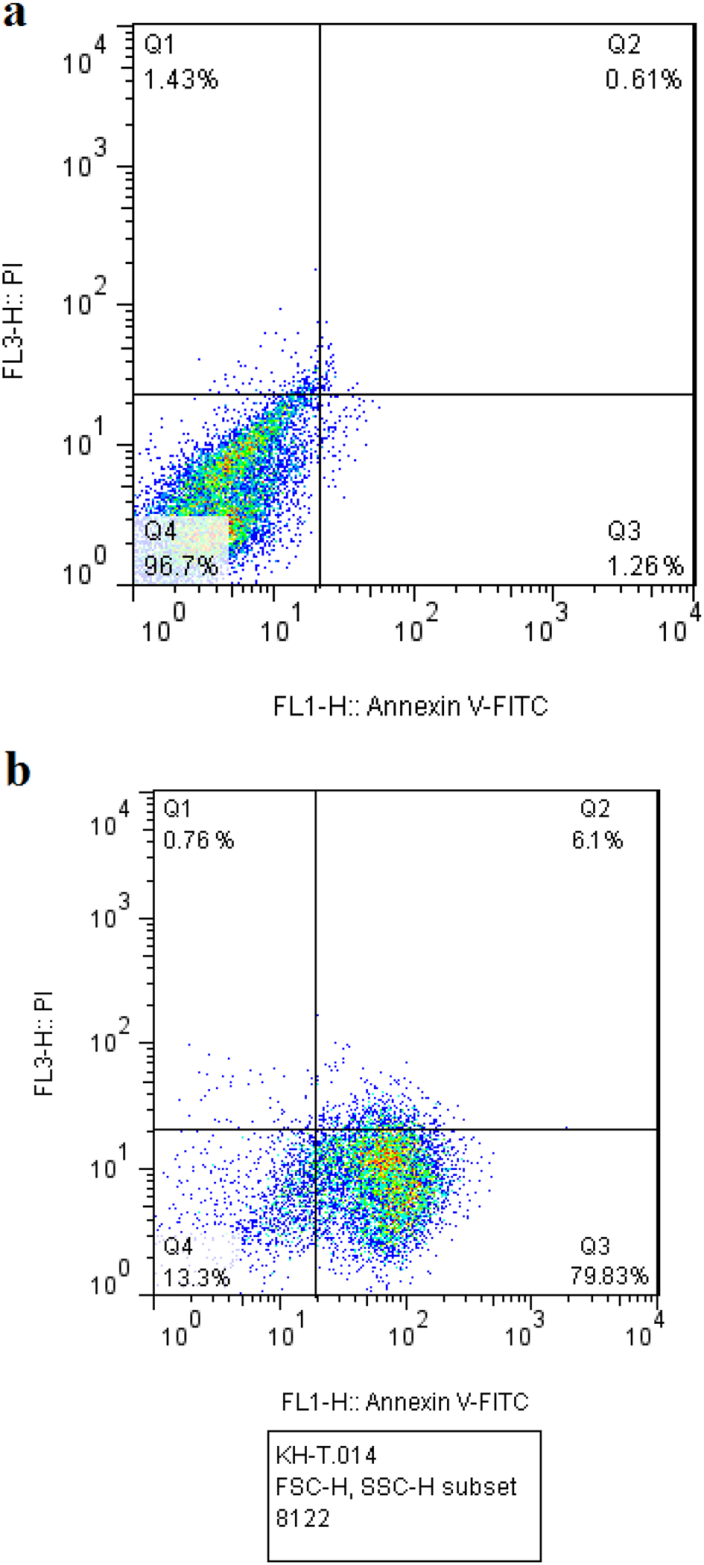
Flow cytometry analysis of the control (**a**) and Ag@Gln-TSC treated cells. According to the results, treating with the nanoparticles considerably increased the frequency of primary apoptosis. Q1; healthy, Q2; Necrosis, Q3; late apoptosis, Q4; early apoptosis.

Flow cytometry analysis of colon cancer cells treated with Ag@Gln-TSC NPs showed that exposure to the nanoparticles caused a remarkable increase in the frequency of primary and late apoptosis. According to the results, the frequency of primary apoptosis in nanoparticle-treated and control cells were 79.83 and 1.26%, respectively, while the frequency of late apoptosis increased from 0.61 to 6.1% (Fig. 9).

**Figure 10:**
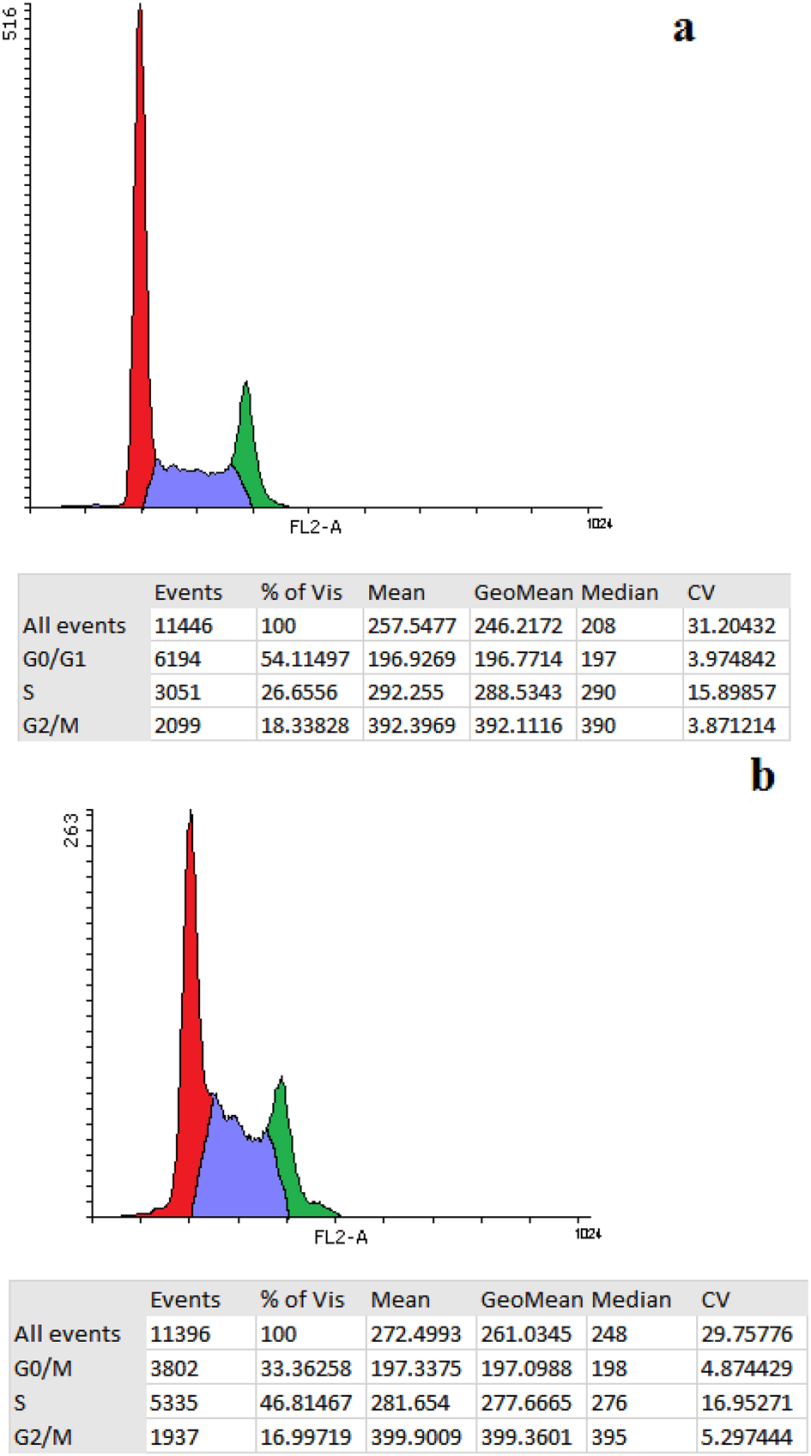
Cell cycle analysis of the control (**a**) and nanoparticle-treated (**b**) cells. The results showed a considerably increase in the population of the cells arrested in the S phase after treating colon cancer cells with Ag@Gln-TSC NPs.

Cell cycle analysis in nanoparticle-treated and control cells showed that in the control group, 54.1% of cells were at the G0/G1 phase, followed by the S phase (26.6%) and G2/M phase (18.3%). In contrast, the frequency of cells at the G0/M, S, and G2/M phases was 33.3, 46.8, and 16.9%, respectively. Overall, the frequency of the cells arrested at the S phase significantly increased by 19.8% (Fig. 10).

### Caspase-3 activity

**Figure 11:**
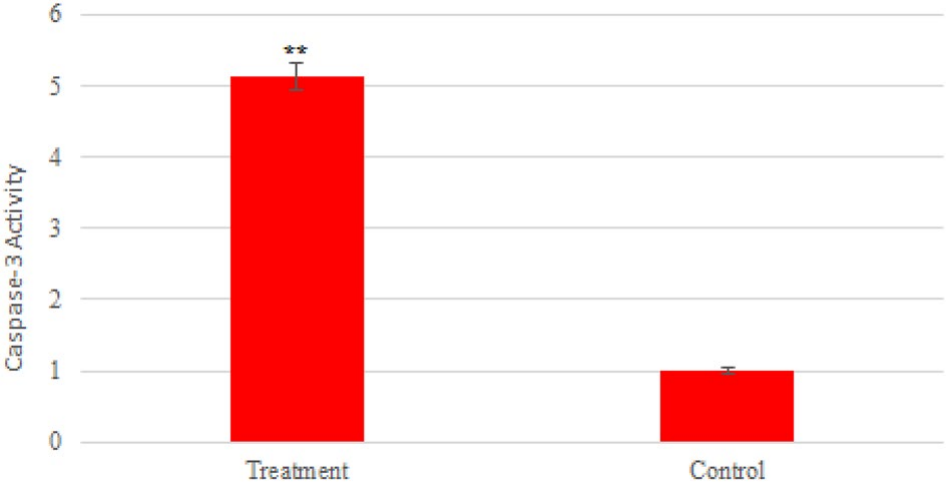
Activity of Caspase-3 in the control and nanoparticle-treated cells. Treating with Ag@Gln-TSC led to a significant increase in the activity of caspase-3 enzyme. Asterisks (*) indicate a significant difference with the control group (**P < 0.01).

The activity of the Caspase-3 was quantified in nanoparticle-treated and control cells. According to the results, after treatment of colon cancer cells with Ag@Gln-TSC NPs, the activity of caspase-3 was increased by 5.2 folds, which was a significant increase compared with the control group. Figure 11 presents the activity of caspase-3 in nanoparticle-treated and control groups.

### Gene expression

**Figure 12:**
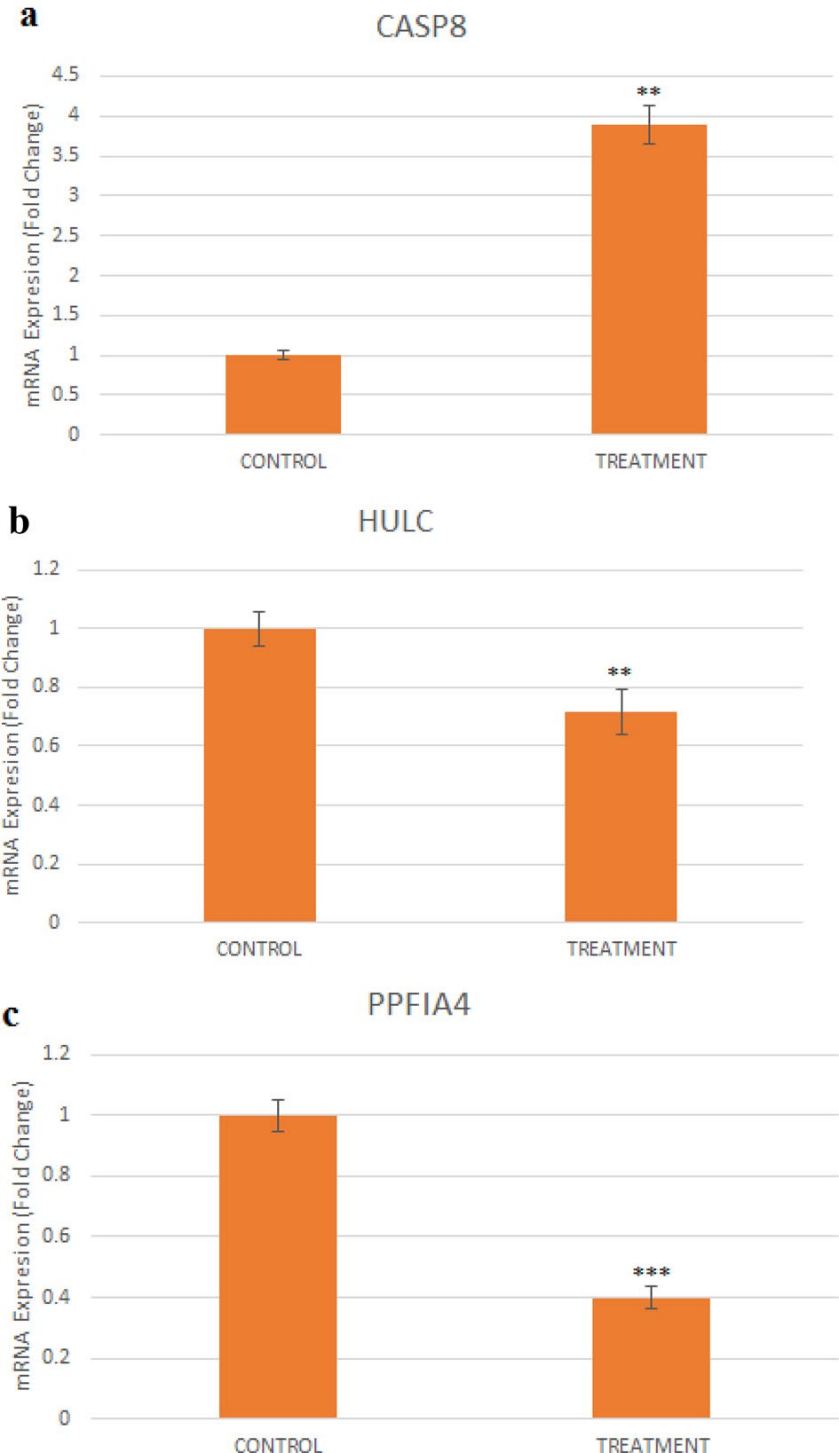
Effect of Ag@Gln-TSC on the expression of the *CASP8* and *PPFIA4* genes and *HULC* LncRNA. The expression of the caspase-8 gene increased significantly, while the HULC LncRNA and PPFIA4 oncogene were considerably down-regulated after treating colon cancer cell with the nanoparticles. Data are normalized to untreated cells and reported as mean ± SD. Asterisks (*) indicate a significant difference with the control group (**P < 0.01; ***P < 0.001).

The effect of Ag@Gln-TSC NP on the expression of the *CASP8*, *HULC*, and *PPIA4* genes was investigated by real-time PCR. According to the results treating colon cancer cells with the nanoparticles significantly increased the expression of the *CASP8* gene by 3.8 folds. In contrast, the expression of the *HULC* Lnc-RNA and *PPIA4* oncogen was significantly reduced to 0.7 and 0.4 folds, in the nanoparticle-treated group. The results were displayed in Fig. 12.

### Hoechst staining

**Figure 13:**
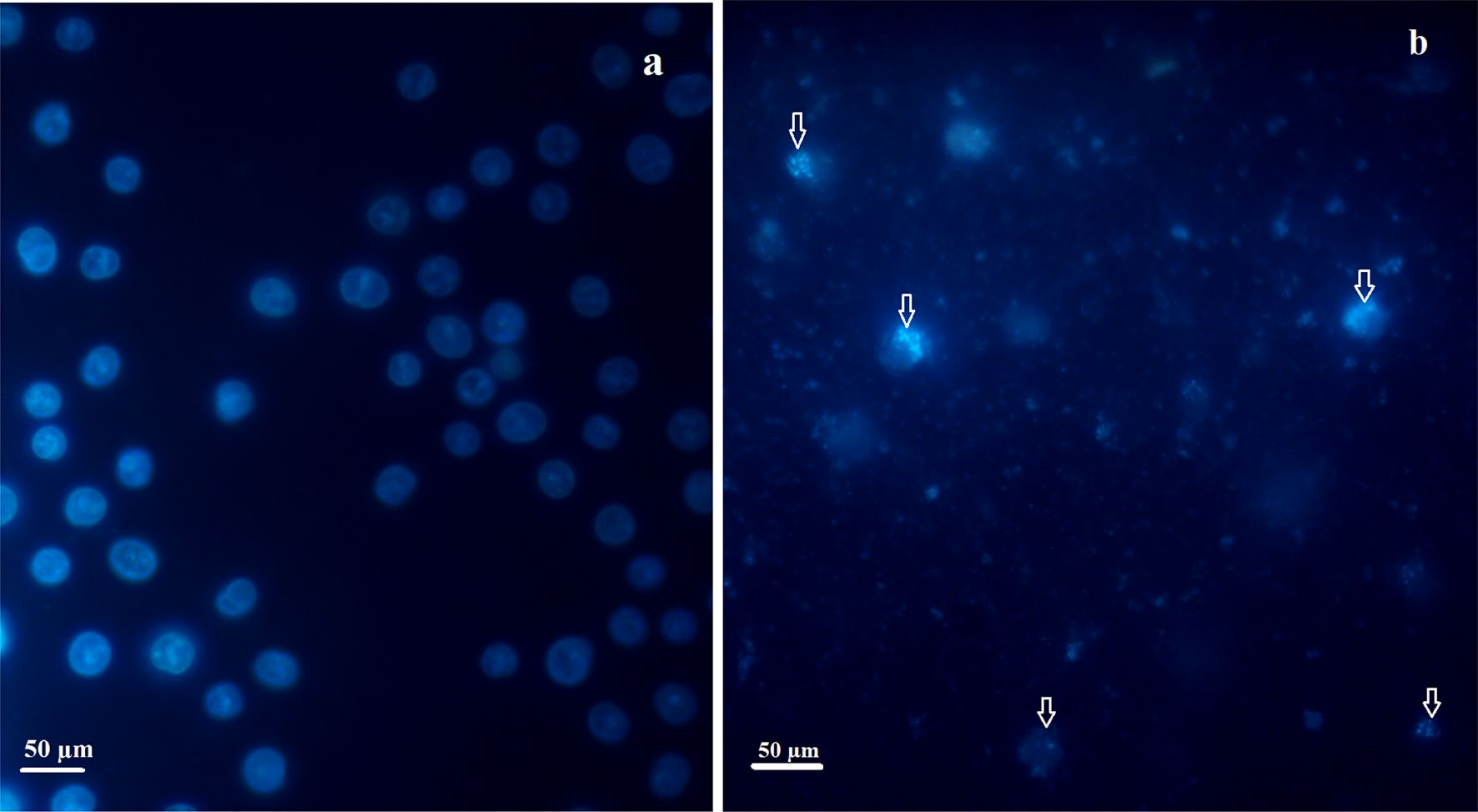
Hoechst staining of colon cancer cells: (**a**) control and (**b**) Ag@Gln-TSC treated. Morphological nuclear alterations, including chromatin fragmentation, chromatin condensation and appearance of apoptotic bodies were noticed in nanoparticle-treated cells.

To evaluate possible nuclear damage caused by Ag@Gln-TSC a Hoechst staining assay was performed. Our results showed that treating colon cancer cells with Ag@Gln-TSC caused considerable nuclear damage which is characterized by chromatin fragmentation, chromosome condensation, and appearance of apoptotic bodies (Fig. 13).

## Discussion

This study aims to synthesize silver nanoparticles functionalized with glutamine and conjugated with thiosemicarbazide and characterize their anticancer feature in a colon cancer cell line. Physicochemical characterization of Ag@Gln-TSC NPs revealed that the particles were synthesized in the nano-scale size range, had no elemental impurity, and were well dispersed. Thermal stability of the particles was confirmed by TGA analysis and FT-IR and XRD patterns confirmed functionalization of silver nanoparticles and conjugation with TSC. Furthermore, the negative surface charge of the particles can provide sufficient repulsive between particles that reduce particle agglomeration.

According to the MTT assay, we found that Ag@Gln-TSC NPs had inhibitory potential for both studied cell lines with a dose-dependent pattern; however, they were remarkably more toxic for SW480 colon cancer cells than HEK293 cell lines, which is a good indicator to show the appropriate anticancer potential and low unwanted toxicity of this substance. Considering the anticancer potential of silver and TSC, which were extensively reported, several mechanisms could be considered for the cytotoxic potential of Ag@Gln-TSC NPs. The mechanism of action of silver nanoparticles in cancer treatment is associated with their ability to induce cell death through several pathways including the generation of ROS molecules that can cause DNA damage, damage to vital biomolecules, and disruption of the cell cycle, which ultimately lead to cell apoptosis in cancer cells^28^. Furthermore, it was reported that silver nanoparticles can induce autophagy of cancer cells through activation of the Ptdlns3K pathway^29^. In addition, it was evident that cancer cells have an enhanced permeation and retention effect which can lead to higher accumulation of silver nanoparticles in cancer cells^30,31^. Therefore, the higher cytotoxicity of Ag@Gln-TSC NPs for colon cancer cells than normal cells may contribute to the enhanced permeability of the nanoparticles into cancer cells.

Functionalization with glutamine can also be considered as another reason for increasing the effectiveness of synthesized nanoparticles on cancer cells compared to normal cells. Glutamine plays a crucial role in human cells, serving as a source of energy and building block for proteins and nucleotides, which is essential for the growth and proliferation of cells. In addition to improving biocompatibility, the functionalization of silver nanoparticles may contribute to improved internalization of the nanoparticles into cancer cells. Cancer cells are characterized by their high proliferation rate which causes higher nutrient demand compared to normal cells. The metabolic requirements of cancer cells to glutamine could be explored to develop targeted therapies that lead to improved efficacy and minimize harm to healthy cells^32^. Due to the higher demand of cancer cells for crucial nutrients, including amino acids, the functionalization of silver nanoparticles with glutamine could improve drug permeability into cancer cells.

Furthermore, the cytotoxic potential of Ag@Gln-TSC NPs is associated with its TSC content as well. It has been reported that TSCs can inhibit cancer cells through interruption of iron intake by cancer cells, generation of oxidative stress, as well as inhibition of DNA replication, which leads to disruption of the cell cycle and induces cell apoptosis^13,14^. Therefore, the anticancer effect of Ag@Gln-TSC NPs seems to be associated with their all constituents, including silver nanoparticles through the generation of oxidative stress, glutamine through improving intracellular nanoparticle penetration, and TSC through various mechanisms. Measuring ROS levels in nanoparticle-treated and control cells showed that treating with Ag@Gln-TSC NPs leads to a remarkable increase in intracellular ROS levels, which represents the generation of oxidative stress as a key anticancer mechanism of the synthesized nanoparticles.

In order to investigate the molecular mechanism of cell death in colon cancer cells due to exposure to nanoparticles, various assays including caspase-3 activity, cell cycle analysis, and frequency of apoptotic cells as well as Hoechst staining were performed. According to our results, treating with Ag@Gln-TSC NPs caused a significant increase in the frequency of apoptotic cells and cell cycle arrest at the S phase. As described above, the generation of oxidative stress and subsequently DNA damage and inhibition of DNA replication could result in cell cycle arrest and apoptosis induction. Previous studies reported that treating with silver nanoparticles can induce DNA damage and cell apoptosis in colon cancer cells through up-regulation of proapoptotic genes^33^. Furthermore, apoptogenic properties and cell cycle arrest by various thiosemicarbazone derivatives in various cancer cells, including colorectal cell lines have been reported in the literature, which is in agreement with our findings^34,35^.

Apoptogenic properties of Ag@Gln-TSC NPs in colon cancer cells were further investigated by investigating caspase-3 activity and Hoechst staining. Our findings revealed that treating with the nanoparticles leads to considerably increased activity of caspase-3, chromatin fragmentation, and formation of nuclear apoptotic bodies which implies the apoptogenic effect of Ag@Gln-TSC NPs in colon cancer cells. Caspase-3 is an enzyme that when activated, plays s central role in initiating a proteolytic cascade leading to cell death through the apoptotic pathway^36^. Hyper-activation of caspase-3 following treatment of colon cancer cells with Ag@Gln-TSC NPs indicates that the induction of apoptosis is the key mechanism in the anticancer effect of the synthesized nanoparticles. Caspase-8 is another key enzyme that is specifically involved in the initiation of extrinsic apoptosis. The extrinsic apoptosis pathway is induced in response to extracellular signals which leads to the activation of the caspase cascade through the activation of the proteolytic activity of caspase-8^37^. Our study showed that Ag@Gln-TSC NPs significantly up-regulated the expression of the *CASP8* gene in the colon cancer cell line. Due to the critical role of caspase-8 in activation of the extrinsic apoptosis, increased expression of the *CASP8* indicates that the activation of extrinsic apoptosis pathway in these cells can be considered. Furthermore, the expression of the PPFIA1 in nanoparticle-treated cells was considerably decreased. PPFIA4 is a cellular oncogene that is involved in promoting the proliferation and migration of various cancer cells, including colon cancer. Up-regulation of the PPFIA4 is correlated with higher clinical stages and poor survival of colon cancer patients^38^. Due to the important role of this oncogene in cancer initiation and development, targeting PPFIA4 in cancer treatment studies can be considered. Our work indicated that PPFIA4 was significantly down-regulated by Ag@Gln-TSC NPs, indicating their potential to inhibit the proliferation of colon cancer cells.

The effect of Ag@Gln-TSC NPs on the expression of *HULC* long non-coding RNA (Lnc-RNA) in colon cancer cells was also investigated. The *HULC* is an Lnc-RNA that is associated with the development of various cancers. It was reported that *HULC* expression is increased in colorectal cancers and is involved with accelerated growth of colon cancer cells through targeting miR-613^39^.

## Conclusion

In this work, Ag@Gln-TSC NPs were synthesized and their anticancer properties in a colon cancer cell line were investigated. Our study revealed that treating colon cancer cells with Ag@Gln-TSC NPs caused significant inhibition of cell proliferation and apoptosis induction through the generation of oxidative stress. Furthermore, it caused considerable expression and activity of caspases as well as down-regulation of the *PPFIA4* oncogene and HULC Lnc-RNA. Our findings indicate that Ag@Gln-TSC is a potent anti-proliferative substance that can be further investigated for anticancer chemotherapy.

## Data Availability

All data generated or analyzed during this study are included in this published article.
